# Tick-Borne Rickettsioses in the Iberian Peninsula

**DOI:** 10.3390/pathogens11111377

**Published:** 2022-11-18

**Authors:** Leonardo Moerbeck, Ana Domingos, Sandra Antunes

**Affiliations:** Global Health and Tropical Medicine, Instituto de Higiene e Medicina Tropical, Universidade Nova de Lisboa (GHTM-IHMT-UNL), Rua da Junqueira, 100, 1349-008 Lisboa, Portugal; moerbeck.leonardo@gmail.com (L.M.); santunes@ihmt.unl.pt (S.A.)

**Keywords:** tick-borne rickettsioses, *Rickettsia* spp., Mediterranean Spotted-Fever (MSF), Iberian Peninsula, surveillance

## Abstract

Tick-borne rickettsioses (TBR) are caused by obligate, intracellular bacteria of the spotted-fever group (SFG) of the genus *Rickettsia* (Order *Rickettsiales*), transmitted by hard ticks. TBR are one of the oldest known vector-borne zoonoses and pose a threat to both human and animal health, as over the years, new SFG *Rickettsia* spp. have been reported worldwide with the potential to be human pathogens. In Portugal and Spain, the countries that constitute the Iberian Peninsula, reported TB rickettsiae causing human disease include *Rickettsia conorii conorii*, *Rickettsia conorii israelensis*, *Rickettsia slovaca*, *Rickettsia raoultii*, *Candidatus* Rickettsia rioja, *Rickettsia sibirica mongolitimonae*, and *Rickettsia monacensis*. An allochthonous case of TBR caused by *Rickettsia massiliae*, described in Spain, points to the need to monitor disease epidemiology, to predict risks of exposure and spread of disease, and taking into account globalization and climate changes. This review aims to provide up-to-date information on the status of TBR in the Iberian Peninsula, as well as to show the importance of a national and international collaborative epidemiology surveillance network, towards monitoring *Rickettsia* spp. circulation in both Portugal and Spain.

## 1. Introduction

Most of the rickettsial species are nonpathogenic endosymbionts circulating in a wide range of organisms (arthropods, protists, and other eukaryotes). Humans are not common hosts for *Rickettsia*, an obligate, intracellular, Gram-negative, α-proteobacteria; however, the recognized pathogenicity in humans of some species has put *Rickettsia* in the spotlight as a public health concern.

With the advent of molecular approaches, the genus *Rickettsia* is currently classified into four major groups: the typhus group (TG), containing *Rickettsia prowazekii* and *Rickettsia typhi*; the ancestral group (AG), formed by *Rickettsia bellii* and *Rickettsia canadensis*; the transitional group (TRG), formed by *Rickettsia akari* and *Rickettsia felis;* and the spotted-fever group (SFG) [1]. Recently, Murray and colleagues [2] reconstructed the *Rickettsia* phylogeny using whole-genome data, describing the evolutionary history of the *Rickettsia* core genome, supporting previous groups (TG, TRG, SFG) and expanding to a few more groups, such as the Bellii and the Canadensis groups.

Human rickettsiosis is a widespread zoonosis that is transmitted by arthropods (lice, fleas, ticks, and other mites). The body louse, *Pediculus humanus corporis* is associated with *R. prowazekii*, the causative agent of epidemic typhus [3]. Concerning the bacteria, *R. typhi*, it can be transmitted to humans when in contact with infected *Xenopsylla cheopis* and *Ctenocephalides felis* [4]. *R. akari* is the etiological agent of rickettsialpox transmitted by the bite of the house mice mite *Liponyssoides sanguineus* [5]. Tick-borne rickettsiosis (TBR) belongs to the spotted-fever group (SFG) of the genus *Rickettsia* (family *Rickettsiaceae*; order *Rickettsiales*) [6] and is the principal source of infections naturally transmitted by ticks in Europe [6,7] and, therefore, the focus of the present review.

**Figure 1:**
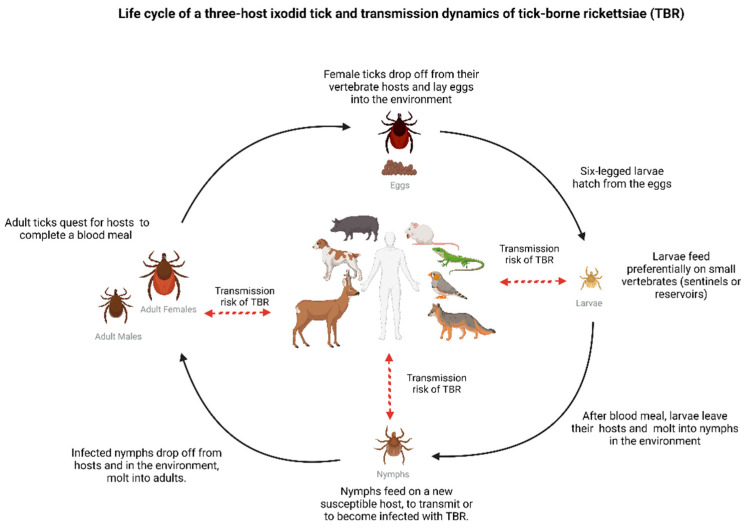
Illustration of a three-host ixodid tick life cycle, depicting the risk of infection and transmission of tick-borne rickettsioses (TBR) at each tick stage [20]. During feeding, ticks use the same canal to (1) inject saliva into the host, enabling transmission of *Rickettsia*, and (2) to acquire blood, providing an entry door for bacteria dissemination. Once reaching the tick midgut, *Rickettsia* may, depending on factors such as bacterial load and time of feeding, propagate within tick tissues, eventually reaching the ovaries (in adult females) and salivary glands (larvae, nymphs, adults) [10,11]. Transstadial transmission of *Rickettsia* promotes maintenance of infection in the tick population and transovarial transmission (reported in many *Rickettsia* spp.) ensures propagation to the next tick generation [6,8]. It is important to note that not all vertebrate hosts present the same potential to maintain infection in nature or tick populations. For example, humans are susceptible hosts to infection but considered dead-end hosts, and ticks do not usually feed on humans [6]. Created with BioRender.com (accessed on 24 October 2022).

Ticks are obligate hematophagous ectoparasites that need, during their post-embryonic stages (larvae, nymphs, and adult stages), at least one vertebrate host (amphibians, reptiles, birds, or mammals) to complete their life cycle. It has been demonstrated that ticks are ubiquitous arthropods, and their diversity is greater in the tropical and subtropical regions of the world [8]. The blood feeding of ticks enables both the transmission and acquisition of a variety of microorganisms, including pathogenic ones. In particular, hard ticks (Ixodidae) present an extended period of feeding that influences the chance of becoming infected and transmitting pathogens, while soft ticks (Argasidae) present a shorter feeding period but feed repeatedly when molting into different nymphal stages. However, pathogens also play a role in this complex equation of transmission/acquisition. It has been reported that some tick-borne viruses are transmissible within minutes (15–60 min), while some bacterial agents, such as *Rickettsia*, require 3–24 h, and protozoans between 24–48 h, of efficient feeding [9]. During the process of feeding, ticks inject saliva and eat blood using the same canal. Hence, pathogens existing in the tick saliva can be passed to the host dermis and blood capillaries and vice-versa: existing pathogens circulating in the vertebrate host can be ingested by the tick. Once pathogens reach the tick’s midgut, the digestive epithelium can be crossed, and pathogens may invade the haemocoel and disseminate to other tick tissues, including the salivary glands. At this point, pathogens can now be injected to a new host via saliva [10,11]. Transovarial (adult female tick to egg) and transstadial transmission (egg to larva to nymph to adult tick) are also important features that make ticks impressive vectors [8], increasing the possibility of transmission. Hard ticks have not only been implicated as natural vectors of TBR, but also as a potential reservoir or amplifiers of these bacteria (illustrated in Figure 1) [6,12]. Even though the reports of soft ticks infected by SFG rickettsiae are increasing [13,14,15,16], there are still no accounts of human cases associated with soft tick bites [13]. Moreover, the role and implications of soft ticks related to TBR transmission deserves further study, as some of the identified *Rickettsia* are pathogenic, e.g., *R. felis* [17,18], and some of the soft tick species from which the bacteria were identified are often found to feed in humans, e.g., *Carios capensis* and *Ornithodoros moubata* [12,19].

As with other vector-borne pathogens, the distribution of TB rickettsiae in nature is directly influenced by the tick’s lifecycle, vector competence, vertebrate hosts availability, and the maintenance of pathogenic microorganisms, e.g., transovarian transmission capacity [6]. For instance, in the Iberian Peninsula, most human cases of Mediterranean spotted-fever (MSF) (*Rickettsia conorii* and subspecies *conorii* and *israelensis*) occur between July and September, coinciding with the high activity of its natural vector, *Rhipicephalus sanguineus* sensu lato (s.l.) and also with the warmer weather that promotes human outdoor activities. This tick vector presents features such as being highly adapted to live within human dwellings and being the most frequent tick infesting dogs worldwide, which in consequence augments human exposure and the risk of bites and transmission of pathogens [21]. Adding to the complexity of the pathogen–vector–host interactions, the environment, landscape alterations, and climate changes can also shape the distribution of TB rickettsiae, by altering the distribution pattern of host and ticks [22,23,24]. In this sense, geographic areas with similar characteristics can be grouped, to better understand TBR epidemiology.

The Iberian Peninsula is primarily composed of Portugal and Spain, separated from the rest of Europe by the Pyrenees and from Africa by the Strait of Gibraltar; therefore, representing a well-delimited geographic region. With a population of about 57 million, both countries share a discrepancy in population density between regions, with lower populations correlating with more rural areas [25], which having in mind the theme of tick-borne diseases should influence the number of people exposed to ticks and at higher risk of contracting disease. Portugal and Spain share land borders, with no great restrictions to wild animal movements. Thus, vertebrate pathogens and vectors easily transit between nations, calling for a collaborative effort to tackle rickettsiosis and other tick-borne menaces. Members of the European Union (EU) are obliged to report certain infectious diseases, including some vector-borne diseases. Aiming for the development of strategies for early detection, prevention, and preparedness, EU policies have led to the establishment of a program for combatting vector-borne diseases in Europe, coordinated by the European Centre for Disease Prevention and Control (ECDC) and the European Food Safety Authority (EFSA) [26]. Surveillance systems and national health authorities are responsible for articulation with these authorities; however, surveillance activities vary among countries, hampering the implementation of local and broad measures to control these challenging groups of diseases [27]. Both Portugal and Spain conduct extensive and active monitoring control of ticks and tick-borne pathogens, managed by national surveillance networks: “REVIVE—Rede de Vigilância de Vetores”, in Portugal; and the national epidemiological surveillance network, “RENAVE—Red Nacional de Vigilancia Epidemiológica”, in Spain [28,29]. While REVIVE focuses on entomological surveillance, the aim of RENAVE is the surveillance of communicable diseases, including TBR. Altogether, these initiatives allow the systematic collection of epidemiological information, its analysis and interpretation, as well as the dissemination of the results across the population and national health agencies, ultimately contributing to the planning of the health research agenda and acting in the evaluation of the biological risk of an emergency in public health [30,31]. In addition, the ECDC regularly publishes maps with the distributions of ticks in Europe, providing an up-to-date distribution and expansion of tick vectors associated with rickettsioses, which highlights the importance of monitoring, not only the pathogens, but also the vectors [32].

Regarding TBR diagnosis, IFA (immunofluorescence assays) are the standard tests. Blood samples should be collected first in the early stage of the disease, and a second sample should be taken two weeks later. A four-fold rise should be obtained. If not, a third sample should be considered after four to six weeks [28,29,32,33,34]. PCR-based molecular tools are also implemented for sensitive and a specific detection and identification of *Rickettsia* spp. in different types of samples, including arthropods tissues. Antimicrobial TBR therapy based on doxycycline remains the standard treatment for these infections, but fluoroquinolones can be considered as an alternative [6]. Clinical practice advice (microscopy, serology, molecular tools, and culture) for the study of *Rickettsia* can be found in a 2017 review [34].

In the present review, TBR will be described according to its prevalence in the Iberian Peninsula, underscoring the need for continuous clinical and entomological vigilance, to assess disease transmission risks. This information can leverage the topic of TBR in the health institutions of both countries and instigate the development of public engagement strategies, to prevent TBR and also other, less prevalent tick-borne diseases (TBD). The keywords used on the PubMed searching tool to obtain up-to-date information regarding the topic were: “*rickettsia*”, “rickettsioses”, “tick”, “tick-borne diseases”, “tick-borne pathogens”, “tick-borne rickettsioses”, “spotted-fever”, “Portugal” and “Spain”.

### 1.1. Tick-Borne Rickettsioses in Europe and in the Iberian Countries

The circulation of *Rickettsia* spp. causing TBR in Europe has been well described, including reports of *R. conorii* transmitted by ticks belonging to *R. sanguineus* complex; *R. helvetica* and *R. monacensis* by *Ixodes ricinus*; *R. slovaca, R. raoultii* and *Candidatus* Rickettsia rioja by *Dermacentor marginatus*; *R. aeschlimannii* by *Hyalomma* spp. and *Rhipicephalus* spp. and *R. sibirica mongolotimonae*, whose involved vectors are no less than *Hyalomma* spp. and *Rhipicephalus pusillus* [6,35] and in the case of *R. massiliae*, apart from its allochthonous case, ticks infected with this SFG *Rickettsia* were: *R. pusillus* [36], *R. sanguineus* s.l. [37,38,39,40,41,42] and *R. turanicus* [43,44,45].

According to ECDC, most cases of rickettsioses are reported from Italy, Portugal, and Spain [35].

### 1.2. Mediterranean Spotted-Fever (MSF)

Mediterranean Spotted-Fever (MSF) was first described in 1910 by Conor and Brunch, in Tunisia and later, in 1923, described by Delfim Pinheiro in Portugal [46]. The causative agent of MSF is *R. conorii*, which encompasses a complex of four subspecies, *R. conorii conorii*, *R. conorii caspia*, *R. conorii israelensis* and *R. conorii indica* [47]. To date, *R. conorii conorii* and *R. conorii israelensis* are the subspecies reported in the Iberian Peninsula, with human cases associated to *R. conorii conorii* in Spain and human cases associated to *R. conorii israelensis* in Portugal [6].

The tick *R. sanguineus* s.l. is recognized as the main vector of the MSF. The subspecies *R. conorii israelensis* have been successfully isolated from a *R. sanguineus* collected in Portugal [48]. This tick species can also be considered a reservoir of *R. connorii* in the Mediterranean region due to transstadial and transovarian transmission that maintain the infection in the tick population [49]. There is still some debate regarding this subject since early studies have demonstrated the persistence of *R. conorii* along tick generations but in nature low infection prevalence in ticks are reported, suggesting that ticks pay a fitness “toll” when infected by this rickettsiae species [11]. Such observations have made researchers speculate about the role of vertebrate hosts as potential reservoirs. In nature, levels of infection of vertebrates are low and are often transient, which does not support maintenance of infection in these hosts. However, there is experimental evidence that dogs can act as reservoirs of *R. conorii*, as dogs became infected by inoculation or after feeding of infected ticks, naïve ticks feeding in infected dogs acquire the bacteria (one-month post-infection and even when infection was not detectable by PCR) and are able to transmit *Rickettsia* to their progeny [12,50,51]. Seroprevalence of *Rickettsia* species in dogs from endemic regions (such as the Iberian Peninsula) also sustains this hypothesis [52,53] and the fact that *R. sanguineus* is the “dog tick” should also be considered [21]. Therefore, and even though the role of vertebrates in the perpetuation of TBR remains unclear, it has been accepted that dogs contribute for the persistence of MSF in nature and can act as sentinels of infection in endemic areas [54]. It is important to point out that while other mammals such as lagomorphs or hedgehogs can be affected by MSF, there are no studies focusing on their potential role as reservoirs [6,55]. 

The dependency of MSF transmission on its vector tightly associates the appearance of human cases with the activity of *R. sanguineus* ticks. Consequently, MSF can be considered a summer illness, since most of the cases occur between July and September, overlapping with the peak of questing activity of immature stages of *R. sanguineus* s.l, although there are records of cases diagnosed in February, November, and December [7,46,56]. It has been demonstrated that climate influences host-seeking behavior of ticks, including of *R. sanguineus* [57,58,59]. Noteworthy, a study conducted in 2007 experimentally showed that *R. sanguineus* is more likely to bite humans after exposition to warmer temperatures, as a consequence of an increased aggressivity on host seeking, and thus, resulting in more cases of human rickettsiosis [59]. Adding to the patterns of tick´s activity, summer season also corresponds to holidays, thus to an increase of outdoor activities and consequently a greater chance of encountering questing ticks.

As referred previously, after a tick biting, the bacteria take from 3 to 24 h to be efficiently transmitted and in average, MSF latent period takes about 6 days with an abrupt onset. Clinical conditions are characterized by fever, flu-like symptoms, prostration, maculopapular or petechial rash and eschar at the tick bite site [6,7,48,55,56,60,61]. In addition, severe manifestations such as encephalitis [62] may occur in patients with advanced age, immunocompromised, chronic alcoholism, glucose 6-phosphate dehydrogenase (G6PD) deficiency, inappropriate use of antibiotics, and delayed of diagnostic and treatment [6,48,63].

MSF diagnosis based on clinical, epidemiological, and laboratorial findings in Portugal between 1989 and 2012, has shown 250 positive cases, in which, mortality rate reached 3.6% [46]. In Spain, from 2005 to 2015, there were 1603 notified MSF cases, and 49.5% were confirmed, with no fatal cases occurring during this period [55]. However, regarding death, it is important to denote such indicator is not well documented in many of the reports. According to RENAVE, in 2016, from the 115 notified cases of MSF, 91 were confirmed [64], and in 2017–2018, from the 557 notifications, 473 cases were confirmed, including 18 imported [30]. It is important to refer that in Spain, until 2015, MSF was considered endemic to certain regions and not all cases were reported to RENAVE which may have led to a under recording of MSF cases in the country [64]. In the particular case of patients diagnosed with MSF caused by *R. conorii israelensis*, nausea and vomiting apart from common clinical manifestations already pointed out, were observed [6]. In addition, reports of eschar inoculation were rarely observed. In Portugal, mortality rate of this subspecies reaches 29% when compared to other MSF (3%), from 1994 to 2006 [48,56,61]. Until the present date, there is no report of human infection caused by *R. conorii israelensis* in Spain. Regarding to the enzootic cycle of *R. conorii israelensis*, in southern Portugal, Maia et al, [56] found dogs and cats infected with this SFG rickettsiae, in medical centers, animal shelters, supporting that these pets may act as reservoirs and/or sentinels of this bacteria, as they were asymptomatic during rickettsial infections.

To date, MSF is the most prevalent zoonosis, and it appears to be endemic throughout the Iberian Peninsula. Moreover, positive cases are not only registered in endemic regions, but also in non-endemic areas [46,55].

### 1.3. Dermacentor-Borne Necrosis Erythema Lymphadenopathy (DEBONEL), Tick-Borne Lymphadenopathy (TIBOLA), and Scalp Eschar and Neck Lymphadenopathy after Tick-Bite (SENLAT)

The known causative agents of DEBONEL / TIBOLA or SENLAT rickettsioses are *R. slovaca*, *R. raoultii* and *Ca.*R. rioja. It is the most prevalent tick-borne rickettsioses in Europe, after MSF [7,65]. The main vector of these SFG-rickettsial agents is *D. marginatus* but at least for *R. raoultii*, there’s evidence that *D. reticulatus* can also transmit this bacterium [66] while for the others, it remains a potential vector [63,65]. Clinical manifestations include fever, headache, rash, myalgia, vertigo and persistent asthenia, neck lymphadenopathy, and a necrotic eschar surrounded by a perilesional erythematous halo at the site of the tick attachment [67,68]. As *Dermacentor* spp. usually bite animals with high fur density, these ticks are frequently found on the scalp of humans, thus the most common symptoms observed in patients are alopecia around the tick-bite site, and facial edema [67,69]. When ticks are not located on the scalp, other hairy zones like thorax, arms and even axillae might be spots for tick-bites, and an erythema, similar to the erythema migrans from Lyme borreliosis, typically appears [7].

DEBONEL’s cases are frequently diagnosed during the late fall and winter to mid spring, which is compatible with higher activity period of its vector [65,69]. *D. marginatus* adult ticks are active from late August/September through April/May (extreme cold and snow interrupt activity). July and August are the months where larvae and nymphs have their activity peak, respectively [65,70].

There are more than 200 reported human cases from Spain since 2000 [65,69] and, at least three from Portugal, since 2010 [68]. Furthermore, most recently in Spain, there was an unprecedent report of I. ricinus ticks infected with R. slovaca, R. raoultii and Ca. R. rioja [63]. Although this is the first report of I. ricinus infected with Rickettsia that cause DEBONEL, further studies should be carried out to understand which possible roles this tick species may play either in the maintenance and/or the transmission of these bacteria, and thus, possibly influencing or interfering directly or indirectly, in the epidemic and enzootic cycles [59].

### 1.4. Lymphangitis-Associated Rickettsiosis (LAR)

*Rickettsia sibirica mongolitimonae*, the causative agent of LAR, is a subspecies of *R. sibirica* and was originally isolated from *Hyalomma asiaticum* tick species collected in the Mongolia in 1991 [71], and recognized as responsible for a human infection in France in 1996 [72]. Even though experimental proven vectors are not recognized, in the Iberian Peninsula, this SFG-*rickettsia* was found in *Rhipicephalus bursa*, *R. pusillus* and in *Hyalomma marginatum* from birds in Spain [73,74] while in Portugal it was detected in a *R. pusillus* tick [75,76]. *R. pusillus* is known to infest wild rabbits but it also can be found on wild carnivorous animals, dogs, and domestic cats, and occasionally humans [77]. *R. bursa* ticks are widely distributed throughout the Mediterranean region, it is very possible an underestimation of the pathogen in this tick species. This tick species can parasitize many different mammals such as, cattle, sheep and goats [77,78,79] emphasizing the need to better understand the role of this vector in the transmission and maintenance of LAR. Even being considered a rare infection, the particular and main clinical manifestation, lymphangitis, makes ground for separating LAR from the remaining TBR [80]. Other signs and symptoms include fever, headache, myalgia, rash, and inoculation eschar [81,82,83,84,85,86]. In Spain, until 2011, there were a total of 24 human cases of LAR [85]. In addition, at least, seven more human cases were reported, including two children [83,84,85,86,87,88,89]. Thus far, in Portugal, two confirmed cases of LAR confirm the importance of this disease in the Iberian Peninsula region [75,81].

### 1.5. Mediterranean Spotted Fever-like

*Rickettsia monacensis* also belongs to the SFG, and it was first isolated and identified in Germany, infecting *I. ricinus* ticks [90]. In Portugal and Spain, *R. monacensis* was detected in *I. ricinus* ticks [63,76,91,92], the suspected natural vector. Also, in Portugal there is a report of infected lizard tissues (*Teira dugesii*), suggesting a possible involvement of the reptile in the maintenance of the enzootic cycle and as well as its potential as a reservoir for this rickettsial agent [93]. So far there are no studies confirming the competency of the later tick species to vector *R. monacensis*. However, the frequency of natural infection in *I. ricinus* suggests a role on the maintenance and transmission of the bacteria at least in Europe [94,95]. There are reports of two cases in Spain, whose patients showed general symptoms such as fever, headache and rash of their trunk and extremities. There was no eschar-inoculation at the tick bite sites. Both patients have recovered without sequelae [96]. In Portugal, one recent case has been reported in an elder patient with a background of cardiomyopathy, diabetes mellitus type 2 and alcohol abuse [37]. All affected patients by *R. monacensis* infection were over 59 years old, raising the suspicion that older people may be susceptible to infection by this *Rickettsia* SFG [76,91,93].

*Rickettsia massiliae*, is another human pathogenic SFG-*Rickettsia*, which was first isolated from *R. turanicus* and *R. sanguineus* s.l. ticks in 1990 and 1993, respectively, in France [38]. To date, there has been only four reported human cases in Europe, only one from Spain. However, this case was imported from South America. Patient showed MSF-like symptoms, such as fever, purpuric rash on the upper and lower extremities, and eschar [39]. In Portugal, *R. massiliae* was first detected in 1995 from *R. turanicus* [45]. Most recently, surveillance studies from Portugal, have reported *R. sanguineus* s.l. collected from dogs and from hedgehogs (*Erinaceus europaeus*) infected with this SFG-rickettsiae [97,98]. In addition, dogs and *R. pusillus* were also infected with *R. massiliae* [76,98]. In Spain, *R. sanguineus* s.l. [40,42,92,99,100,101], *R. turanicus* and *R. pusillus* [36] were all found infected with this *Rickettsia* species.

To date, there are no human cases reported in both Iberian countries caused by *Rickettsia helvetica.* However, this SFG *Rickettsia* has been reported infecting lizard tissues (*T. dugesii*) in Portugal [93] as well as its main vector, *I. ricinus*, in both Portugal and Spain [36,92,93]. In addition, this bacterium, was also found in *Ixodes ventalloi* in Portugal [43], a poorly studied and permissive tick species known to parasitize mainly *Oryctolagus cuniculus* but also birds [102]. Patients around Europe have generally shown mild fever, headache, myalgia, and occasionally rash. Isolated case from Sweden, whom patient was immunocompetent, evolved to septicemic fever, myalgias, arthralgias, severe headache and photophobia [103].

Another MSF-like infection is caused by *R. aeschlimannii*, however the first documented human case caused by this SFG *Rickettsia* in Europe was an allochthonous one, as the patient was returning from Africa to his home country, France [103]. No records of autochthonous cases have been reported in the Iberian Peninsula. However, there have been reports of this bacterium in six anthropophilic tick species in Spain (*H. marginatum*, *H. punctata*, *I. ricinus*, *R. bursa*, *R. sanguineus* s.l., and *R. turanicus*) [36,41,74,104,105,106], and infecting *H. marginatum* in Portugal [43].

## 2. Conclusions

Tick-borne rickettsioses pose a serious health threat in the present globalization scenario. Many factors may rely on this matter, such as routes of migratory birds potentially spreading previously infected ticks with TBR from distant geographical regions [74], travelling or migration of asymptomatic individuals, from an endemic area to a non-endemic area [39], where potentially vectors are available [39]. Behaviors linked to a more healthy and sustainable livings have led to an increment of ecotourism, trails, hiking and outdoor activities, thus increasing the risk of tick bites. Moreover, and ultimately, an ordinary, but worrying habit, which non stray dogs circulate between wild and anthropophilic environments, consequently becoming carriers of infected ticks. In this later scenario, dogs may act as the primary vertebrate host for a possible triggering situation of an epidemic TBR´s cycle [107]. In regard to tick and *Rickettsia* spp. relationship, seasonality and availability of tick population is another factor that impacts towards the dynamics of TBR prognosis and diagnosis, as depicted here with MSF and DEBONEL/TIBOLA/SENLAT [6,60,67,73,108,109]. Another matter of great concerning is about companion animals, not only dogs and cats, but also some unconventional pets that have become more popular nowadays, like mammals from the superfamily Musteloidea, including (*Mustela* spp. and *Mephitis* spp.) [110]. In addition to all these, climate and environmental changes may affect not only the distribution of vectors, but also the availability of their vertebrate hosts worldwide, thus, increasing the awareness about TBRs. Moreover, studies that address tick surveillance are crucial for the understanding of how the environment, ticks, and vertebrate hosts intertwine and affect the enzootic, and sometimes, the epidemic cycles of *Rickettsia* spp.

As some SFG *Rickettsia* are of unknown pathogenicity to humans, it is of utmost importance that both countries maintain a network of epidemiology surveillance active, towards monitoring new human cases as it assists in the flow of information on underreported cases and facilitates more accurate diagnosis. Even having in mind that countries present important socio-economic differences, efforts should be made to harmonize protocols, sampling strategies and initiatives, since countries are bounded by similar constraints concerning tick-borne diseases, making particularly important the implementation of similar policies. Such will certainly improve effectiveness in disease prevention and management.

Lastly, actions provided by physicians, veterinarians and researchers to educate, and to spread scientific and useful information about the risks and prevention of tick bites and TBR transmission to citizens, patients, pet owners and outdoor enthusiasts are extremely important, not only to notify public agencies, but also to establish an intersectoral network to exchange information. Such understanding would be of great value, as prevention mostly relies on public health education, besides it would strengthen the One Health approach.

## References

[1] Gillespie J.J., Williams K., Shukla M., Snyder E.E., Nordberg E.K., Ceraul S.M., Dharmanolía C., Rainey D., Soneja J., Shallom J.M. (2008). *Rickettsia* Phylogenomics: Unwinding the Intricacies of Obligate Intracellular Life. PLoS ONE.

[2] Murray G.G.R., Weinert L.A., Rhule E.L., Welch J.J. (2016). The Phylogeny of *Rickettsia* Using Different Evolutionary Signatures: How Tree-Like Is Bacterial Evolution?. Syst. Biol..

[3] Akram S.M., Ladd M., King K.C. (2022). Rickettsia Prowazekii. StatPearls.

[4] Blanton L.S., Dumler J.S., Walker D.H. (2014). *Rickettsia typhi* (Murine Typhus). Mandell Douglas Bennett’s Princ. Pract. Infect. Dis..

[5] Akram S.M., Jamil R.T., Gossman W. (2022). Rickettsia akari. StatPearls.

[6] Parola P., Paddock C.D., Socolovschi C., Labruna M.B., Mediannikov O., Kernif T., Abdad M.Y., Stenos J., Bitam I., Fournier P.E. (2013). Update on Tick-Borne Rickettsioses around the World: A Geographic Approach. Clin. Microbiol. Rev..

[7] Oteo J.A., Portillo A. (2012). Tick-Borne Rickettsioses in Europe. Ticks Tick Borne Dis..

[8] Sonenshine D.E., Roe M., Sonenshine D.E., Roe R.M. (2014). Ticks, People and Animals. Biology of Ticks.

[9] Tahir D., Meyer L., Fourie J., Jongejan F., Mather T., Choumet V., Blagburn B., Straubinger R.K., Varloud M. (2020). Interrupted Blood Feeding in Ticks: Causes and Consequences. Microorganisms.

[10] de la Fuente J., Antunes S., Bonnet S., Cabezas-Cruz A., Domingos A.G., Estrada-Peña A., Johnson N., Kocan K.M., Mansfield K.L., Nijhof A.M. (2017). Tick-Pathogen Interactions and Vector Competence: Identification of Molecular Drivers for Tick-Borne Diseases. Front. Cell Infect. Microbiol..

[11] Spernovasilis N., Markaki I., Papadakis M., Mazonakis N., Ierodiakonou D. (2021). Mediterranean Spotted Fever: Current Knowledge and Recent Advances. Trop. Med. Infect. Dis..

[12] Tomassone L., Portillo A., Nováková M., de Sousa R., Oteo J.A. (2018). Neglected Aspects of Tick-Borne Rickettsioses. Parasit. Vectors.

[13] Milhano N., Palma M., Marcili A., Núncio M.S., de Carvalho I.L., de Sousa R. (2014). *Rickettsia lusitaniae* sp. Nov. Isolated from the Soft Tick *Ornithodoros erraticus* (Acarina: Argasidae). Comp. Immunol. Microbiol. Infect. Dis..

[14] Chitanga S., Chambaro H.M., Moonga L.C., Hayashida K., Yamagishi J., Muleya W., Changula K., Mubemba B., Simbotwe M., Squarre D. (2021). *Rickettsia lusitaniae* in *Ornithodoros porcinus* Ticks, Zambia. Pathogens.

[15] Qiu Y., Simuunza M., Kajihara M., Chambaro H., Harima H., Eto Y., Simulundu E., Squarre D., Torii S., Takada A. (2021). Screening of Tick-Borne Pathogens in Argasid Ticks in Zambia: Expansion of the Geographic Distribution of *Rickettsia lusitaniae* and *Rickettsia hoogstraalii* and Detection of Putative Novel *Anaplasma* Species. Ticks Tick Borne Dis..

[16] Palomar A.M., Veiga J., Portillo A., Santibáñez S., Václav R., Santibáñez P., Oteo J.A., Valera F. (2021). Novel Genotypes of Nidicolous *Argas* Ticks and Their Associated Microorganisms From Spain. Front. Vet. Sci..

[17] Reeves W.K., Loftis A.D., Sanders F., Spinks M.D., Wills W., Denison A.M., Dasch G.A. (2006). *Borrelia, Coxiella*, and *Rickettsia* in *Carios capensis* (Acari: Argasidae) from a Brown Pelican (*Pelecanus occidentalis*) Rookery in South Carolina, USA. Exp. Appl. Acarol..

[18] Pérez-Osorio C.E., Zavala-Velázquez J.E., León J.J.A., Zavala-Castro J.E. (2008). *Rickettsia felis* as Emergent Global Threat for Humans. Emerg. Infect. Dis..

[19] Estrada-Peña A., Jongejan F. (1999). Ticks Feeding on Humans: A Review of Records on Human-Biting Ixodoidea with Special Reference to Pathogen Transmission. Exp. Appl. Acarol..

[20] Centers for Disease Control and Prevention. https://www.cdc.gov/.

[21] Dantas-Torres F. (2010). Biology and Ecology of the Brown Dog Tick, *Rhipicephalus sanguineus*. Parasit. Vectors.

[22] Mysterud A., Jore S., Østerås O., Viljugrein H. (2017). Emergence of Tick-Borne Diseases at Northern Latitudes in Europe: A Comparative Approach. Sci. Rep..

[23] Gilbert L. (2021). The Impacts of Climate Change on Ticks and Tick-Borne Disease Risk. Annu. Rev. Entomol..

[24] Semenza J.C., Suk J.E. (2018). Vector-Borne Diseases and Climate Change: A European Perspective. FEMS Microbiol. Lett..

[25] Península Ibérica Em Números 2020-The Iberian Peninsula 2020. https://www.ine.pt/ine_novidades/PIN2020/.

[26] Combatting Vector-Borne Diseases in Europe: EFSA and ECDC. https://www.ecdc.europa.eu/en/publications-data/combatting-vector-borne-diseases-europe-efsa-and-ecdc.

[27] Garcia-Vozmediano A., de Meneghi D., Sprong H., Portillo A., Oteo J.A., Tomassone L. (2022). A One Health Evaluation of the Surveillance Systems on Tick-Borne Diseases in the Netherlands, Spain and Italy. Vet. Sci..

[28] Rede de Vigilância de Vetores | REVIVE Categoria-INSA. https://www.insa.min-saude.pt/category/areas-de-atuacao/doencas-infeciosas/revive-rede-de-vigilancia-de-vetores/.

[29] Fiebre Exantemática Mediterránea. https://www.isciii.es/QueHacemos/Servicios/VigilanciaSaludPublicaRENAVE/EnfermedadesTransmisibles/Paginas/FiebreExantematica.aspx.

[30] Nacional de Epidemiología Instituto de Salud Carlos III Ministerio de Ciencia Innovación (2018). Resultados de La Vigilancia Epidemiológica de Las Enfermedades Transmisibles Informe Anual. Años 2017–2018. https://www.isciii.es/QueHacemos/Servicios/VigilanciaSaludPublicaRENAVE/EnfermedadesTransmisibles/Documents/INFORMES/INFORMES%20RENAVE/RENAVE_Informe_anual__2017-2018.pdf.

[31] Instituto Nacional Doutor Ricardo Jorge (2021). Relatório REVIVE 2020-Culicídeos e Ixodídeos: Rede de Vigilância de Vetores. https://www.insa.min-saude.pt/revive-rede-de-vigilancia-de-vetores-relatorio-2021/.

[32] Tick Maps. https://www.ecdc.europa.eu/en/disease-vectors/surveillance-and-disease-data/tick-maps.

[33] Brouqui P., Bacellar F., Baranton G., Birtles R.J., Bjoërsdorff A., Blanco J.R., Caruso G., Cinco M., Fournier P.E., Francavilla E. (2004). Guidelines for the Diagnosis of Tick-Borne Bacterial Diseases in Europe. Clin. Microbiol. Infect..

[34] Portillo A., de Sousa R., Santibáñez S., Duarte A., Edouard S., Fonseca I.P., Marques C., Novakova M., Palomar A.M., Santos M. (2017). Guidelines for the Detection of *Rickettsia* spp.. Vector Borne Zoonotic Dis..

[35] Rickettsiosis. https://www.ecdc.europa.eu/en/rickettsiosis.

[36] Fernández-Soto P., Pérez-Sánchez R., Álamo-Sanz R., Encinas-Grandes A. (2006). Spotted Fever Group Rickettsiae in Ticks Feeding on Humans in Northwestern Spain: Is *Rickettsia conorii* Vanishing?. Ann. N. Y. Acad Sci..

[37] de Sousa R., dos Santos M.L., Cruz C., Almeida V., Garrote A.R., Ramirez F., Seixas D., Manata M.J., Maltez F. (2022). Rare Case of Rickettsiosis Caused by *Rickettsia monacensis*, Portugal, 2021. Emerg. Infect. Dis..

[38] Beati L., Raoult D. (1993). *Rickettsia massiliae* sp. Nov., a New Spotted Fever Group Rickettsia. Int. J. Syst. Bacteriol..

[39] García-García J.C., Portillo A., Núñez M.J., Santibáñez S., Castro B., Oteo J.A. (2010). A Patient from Argentina Infected with *Rickettsia massiliae*. Am. J. Trop. Med. Hyg..

[40] Merino F.J., Nebreda T., Serrano J.L., Fernández-Soto P., Encinas A., Pérez-Sánchez R. (2005). Tick Species and Tick-Borne Infections Identified in Population from a Rural Area of Spain. Epidemiol. Infect..

[41] Oteo J.A., Portillo A., Santibáñez S., Pérez-Martínez L., Blanco J.R., Jiménez S., Ibarra V., Pérez-Palacios A., Sanz M. (2006). Prevalence of Spotted Fever Group *Rickettsia* Species Detected in Ticks in La Rioja, Spain. Ann. N. Y. Acad Sci..

[42] Beati L., Roux V., Ortuno A., Ortuno O., Castella J., Segura Porta F., Raoult A.D. (1996). Phenotypic and Genotypic Characterization of Spotted Fever Group Rickettsiae Isolated from Catalan *Rhipicephalus sanguineus* Ticks. J. Clin. Microbiol..

[43] Santos-Silva M.M., Sousa R., Santos A.S., Melo P., Encarnação V., Bacellar F. (2006). Ticks Parasitizing Wild Birds in Portugal: Detection of *Rickettsia aeschlimannii*, *R. helvetica* and *R. massiliae*. Exp. Appl. Acarol..

[44] Márquez F.J. (2008). Spotted Fever Group *Rickettsia* in Ticks from Southeastern Spain Natural Parks. Exp. Appl. Acarol..

[45] Bacellar F., Regnery R.L., Núncio M.S., Filipe A.R. (1995). Genotypic Evaluation of Rickettsial Isolates Recovered from Various Species of Ticks in Portugal. Epidemiol. Infect..

[46] Crespo P., Seixas D., Marques N., Oliveira J., da Cunha S., Meliço-Silvestre A. (2015). Mediterranean Spotted Fever: Case Series of 24 Years (1989–2012). Springerplus.

[47] Zhu Y., Fournier P.E., Eremeeva M., Raoult D. (2005). Proposal to Create Subspecies of *Rickettsia conorii* Based on Multi-Locus Sequence Typing and an Emended Description of *Rickettsia conorii*. BMC Microbiol..

[48] de Sousa R., Santos-Silva M., Santos A.S., Barros S.C., Torgal J., Walker D.H., Bacellar F. (2007). *Rickettsia conorii israeli* Tick Typhus Strain Isolated from *Rhipicephalus sanguineus* Ticks in Portugal. Vector Borne Zoonotic Dis..

[49] Parola P., Socolovschi C., Raoult D. (2009). Deciphering the Relationships between *Rickettsia conorii conorii* and *Rhipicephalus sanguineus* in the Ecology and Epidemiology of Mediterranean Spotted Fever. Ann. N. Y. Acad. Sci..

[50] Levin M.L., Killmaster L.F., Zemtsova G.E. (2012). Domestic Dogs (*Canis familiaris*) as Reservoir Hosts for *Rickettsia conorii*. Vector Borne Zoonotic Dis..

[51] Kelly P.J., Matthewman L.A., Mason P.R., Courtney S., Katsande C., Rukwava J. (1992). Experimental Infection of Dogs with a Zimbabwean Strain of *Rickettsia conorii*. J. Trop. Med. Hyg..

[52] Segura-Porta F., Diestre-Ortin G., Ortuño-Romero A., Sanfeliu-Sala I., Font-Creus B., Muñoz-Espin T., de Antonio E.M., Casal-Fábrega J. (1998). Prevalence of Antibodies to Spotted Fever Group Rickettsiae in Human Beings and Dogs from and Endemic Area of Mediterranean Spotted Fever in Catalonia, Spain. Eur. J. Epidemiol..

[53] Alexandre N., Santos A.S., Bacellar F., Boinas F.J., Núncio M.S., de Sousa R. (2011). Detection of *Rickettsia conorii* Strains in Portuguese Dogs (*Canis familiaris*). Ticks Tick Borne Dis..

[54] Ortuño A., Pons I., Nogueras M.M., Castellà J., Segura F. (2009). The Dog as an Epidemiological Marker of *Rickettsia conorii* Infection. Clin. Microbiol. Infect..

[55] Romaní Vidal A., Fernández-Martínez B., Herrador Z., León Gómez I., Gómez Barroso D. (2020). Spatial and Temporal Trends of Mediterranean Spotted Fever in Spain, 2005-2015. Ticks Tick Borne Dis..

[56] Maia C., Cristóvão J.M., Pereira A., Parreira R., Campino L. (2019). Detection of *Rickettsia conorii israelensis* DNA in the Blood of a Cat and a Dog From Southern Portugal. Top. Companion Anim. Med..

[57] Beugnet F., Kolasinski M., Michelangeli P.A., Vienne J., Loukos H. (2011). Mathematical Modelling of the Impact of Climatic Conditions in France on *Rhipicephalus sanguineus* Tick Activity and Density since 1960. Geospat. Health.

[58] Mangan M.J., Foré S.A., Kim H.J. (2022). Seasonal Changes in Questing Efficiency of Wild *Amblyomma americanum* (Acari: Ixodidae) Nymphs. Ticks Tick Borne Dis..

[59] Parola P., Socolovschi C., Jeanjean L., Bitam I., Fournier P.E., Sotto A., Labauge P., Raoult D. (2008). Warmer Weather Linked to Tick Attack and Emergence of Severe Rickettsioses. PLoS Negl. Trop. Dis..

[60] Piotrowski M., Rymaszewska A. (2020). Expansion of Tick-Borne Rickettsioses in the World. Microorganisms.

[61] de Sousa R., França A., Nòbrega S.D., Belo A., Amaro M., Abreu T., Poças J., Proença P., Vaz J., Torgal J. (2008). Host- and Microbe-Related Risk Factors for and Pathophysiology of Fatal *Rickettsia conorii* Infection in Portuguese Patients. J. Infect. Dis..

[62] Mendes J.B., Gomes J.F., Gonçalves T., Canhão B., Madaleno J. (2021). Encephalitis: A Rare Complication of Mediterranean Spotted Fever. IDCases.

[63] Remesar S., Díaz P., Portillo A., Santibáñez S., Prieto A., Díaz-Cao J.M., López C.M., Panadero R., Fernández G., Díez-Baños P. (2019). Prevalence and Molecular Characterization of *Rickettsia* spp. in Questing Ticks from North-Western Spain. Exp. Appl. Acarol..

[64] Nacional de Epidemiología Instituto de Salud Carlos III Ministerio de Ciencia, Innovación y Universidades (2018). Resultados de La Vigilancia Epidemiológica de Las Enfermedades Transmisibles. Informe Anual. Año 2016. http://gesdoc.isciii.es/gesdoccontroller?action=download&id=25/01/2019-d8ee271b6f.

[65] Santibáñez S., Portillo A., Ibarra V., Santibáñez P., Metola L., García-García C., Palomar A.M., Cervera-Acedo C., Alba J., Blanco J.R. (2022). Epidemiological, Clinical, and Microbiological Characteristics in a Large Series of Patients Affected by Dermacentor-Borne-Necrosis-Erythema-Lymphadenopathy from a Unique Centre from Spain. Pathogens.

[66] Földvári G., Rigó K., Lakos A. (2013). Transmission of *Rickettsia slovaca* and *Rickettsia raoultii* by Male *Dermacentor marginatus* and *Dermacentor reticulatus* Ticks to Humans. Diagn. Microbiol. Infect. Dis..

[67] Porta F.S., Nieto E.A., Creus B.F., Espín T.M., Casanova F.J.T., Sala I.S., García S.L., Aguilar J.L., Vilaseca M.Q. (2008). Tick-Borne Lymphadenopathy: A New Infectious Disease in Children. Pediatr. Infect. Dis. J..

[68] de Sousa R., Pereira B.I., Nazareth C., Cabral S., Ventura C., Crespo P., Marques N., da Cunha S. (2013). *Rickettsia slovaca* Infection in Humans, Portugal. Emerg. Infect. Dis..

[69] Silva-Pinto A., de Lurdes Santos M., Sarmento A. (2014). Tick-Borne Lymphadenopathy, an Emerging Disease. Ticks Tick Borne Dis..

[70] Rubel F., Brugger K., Pfeffer M., Chitimia-Dobler L., Didyk Y.M., Leverenz S., Dautel H., Kahl O. (2016). Geographical Distribution of *Dermacentor marginatus* and *Dermacentor reticulatus* in Europe. Ticks Tick Borne Dis..

[71] Yu X., Jin Y., Fan M., Xu G., Liu Q., Raoult D. (1993). Genotypic and Antigenic Identification of Two New Strains of Spotted Fever Group Rickettsiae Isolated from China. J. Clin. Microbiol..

[72] Raoult D., Brouqui P., Roux V. (1996). A New Spotted-Fever-Group Rickettsiosis. Lancet.

[73] Toledo Á., Olmeda A.S., Escudero R., Jado I., Valcárcel F., Casado-Nistal M.A., Rodríguez-Vargas M., Gil H., Anda P. (2009). Tick-Borne Zoonotic Bacteria in Ticks Collected from Central Spain. Am. J. Trop. Med. Hyg..

[74] Palomar A.M., Portillo A., Mazuelas D., Roncero L., Arizaga J., Crespo A., Gutiérrez Ó., Márquez F.J., Cuadrado J.F., Eiros J.M. (2016). Molecular Analysis of Crimean-Congo Hemorrhagic Fever Virus and *Rickettsia* in *Hyalomma marginatum* Ticks Removed from Patients (Spain) and Birds (Spain and Morocco), 2009–2015. Ticks Tick Borne Dis..

[75] de Sousa R., Barata C., Vitorino L., Santos-Silva M., Carrapato C., Torgal J., Walker D., Bacellar F. (2006). *Rickettsia sibirica* Isolation from a Patient and Detection in Ticks, Portugal. Emerg. Infect. Dis..

[76] Santos A.S., de Bruin A., Veloso A.R., Marques C., Pereira da Fonseca I., de Sousa R., Sprong H., Santos-Silva M.M. (2018). Detection of *Anaplasma phagocytophilum*, *Candidatus* Neoehrlichia sp., *Coxiella burnetii* and *Rickettsia* spp. in Questing Ticks from a Recreational Park, Portugal. Ticks Tick Borne Dis..

[77] Santos-Silva M.M., Beati L., Santos A.S., de Sousa R., Núncio M.S., Melo P., Santos-Reis M., Fonseca C., Formosinho P., Vilela C. (2011). The Hard-Tick Fauna of Mainland Portugal (Acari: Ixodidae): An Update on Geographical Distribution and Known Associations with Hosts and Pathogens. Exp. Appl. Acarol..

[78] Ferrolho J., Antunes S., Santos A.S., Velez R., Padre L., Cabezas-Cruz A., Santos-Silva M.M., Domingos A. (2016). Detection and Phylogenetic Characterization of *Theileria* spp. and *Anaplasma marginale* in *Rhipicephalus bursa* in Portugal. Ticks Tick Borne Dis..

[79] Walker J.B., Keirans J.E., Horak I.G. (2000). The Genus Rhipicephalus (Acari, Ixodidae).

[80] Fournier P.E., Gouriet F., Brouqui P., Lucht F., Raoult D. (2005). Lymphangitis-Associated Rickettsiosis, a New Rickettsiosis Caused by *Rickettsia sibirica mongolotimonae*: Seven New Cases and Review of the Literature. Clin. Infect. Dis..

[81] de Sousa R., Duque L., Anes M., Poças J., Torgal J., Bacellar F., Olano J.P., Walker D.H. (2008). Lymphangitis in a Portuguese Patient Infected with *Rickettsia sibirica*. Emerg. Infect. Dis..

[82] Aguirrebengoa K., Portillo A., Santibáñez S., Marín J.J., Montejo M., Oteo J.A. (2008). Human *Rickettsia sibirica mongolitimonae* Infection, Spain. Emerg. Infect. Dis..

[83] Pulido-Pérez A., Gómez-Recuero L., Lozano-Masdemont B., Suárez-Fernández R. (2015). *Rickettsia sibirica mongolitimonae* Infection in Two Immunocompetent Adults. Enferm. Infecc. Y Microbiol. Clin..

[84] Revilla-Martí P., Cecilio-Irazola Á., Gayán-Ordás J., Sanjoaquín-Conde I., Linares-Vicente J.A., Oteo J.A. (2017). Acute Myopericarditis Associated with Tickborne *Rickettsia sibirica mongolitimonae*. Emerg. Infect. Dis..

[85] Ramos J.M., Jado I., Padilla S., Masiá M., Anda P., Gutiérrez F. (2013). Human Infection with *Rickettsia sibirica mongolitimonae*, Spain, 2007–2011-Volume 19, Number 2—February 2013-Emerging Infectious Diseases Journal-CDC. Emerg. Infect. Dis..

[86] Ibarra V., Portillo A., Palomar A.M., Sanz M.M., Metola L., Blanco J.R., Oteo J.A. (2012). Septic Shock in a Patient Infected with *Rickettsia sibirica mongolitimonae*, Spain. Clin. Microbiol. Infect..

[87] Nogueras M.M., Roson B., Lario S., Sanfeliu I., Pons I., Anton E., Casanovas A., Segura F. (2015). Coinfection with “*Rickettsia sibirica* Subsp. *mongolotimonae*” and *Rickettsia conorii* in a Human Patient: A Challenge for Molecular Diagnosis Tools. J. Clin. Microbiol..

[88] Echevarría-Zubero R., Porras-López E., Campelo-Gutiérrez C., Rivas-Crespo J.C., Lucas A.M.D., Cobo-Vázquez E. (2021). Lymphangitis-Associated Rickettsiosis by *Rickettsia sibirica mongolitimonae*. J. Pediatr. Infect. Dis. Soc..

[89] Vázquez-Pérez Á., Rodríguez-Granger J., Calatrava-Hernández E., Santos-Pérez J.L. (2022). Pediatric Tubular Acute Lymphangitis Caused by *Rickettsia sibirica mongolitimonae*: Case Report and Literature Review. Enferm. Infecc. Y Microbiol. Clin..

[90] Simser J.A., Palmer A.T., Fingerle V., Wilske B., Kurtti T.J., Munderloh U.G. (2002). *Rickettsia monacensis* sp. Nov., a Spotted Fever Group *Rickettsia*, from Ticks (*Ixodes ricinus*) Collected in a European City Park. Appl. Environ. Microbiol..

[91] Carvalho I.L.D., Milhano N., Santos A.S., Almeida V., Barros S.C., de Sousa R., Núncio M.S. (2008). Detection of *Borrelia lusitaniae*, *Rickettsia* sp. IRS3, *Rickettsia monacensis*, and *Anaplasma phagocytophilum* in *Ixodes ricinus* Collected in Madeira Island, Portugal. Vector Borne Zoonotic Dis..

[92] Palomar A.M., Santibáñez P., Mazuelas D., Roncero L., Santibáñez S., Portillo A., Oteo J.A. (2012). Role of Birds in Dispersal of Etiologic Agents of Tick-Borne Zoonoses, Spain, 2009. Emerg. Infect. Dis..

[93] de Sousa R., de Carvalho I.L., Santos A.S., Bernardes C., Milhano N., Jesus J., Menezes D., Núncio M.S. (2012). Role of the Lizard *Teira dugesii* as a Potential Host for *Ixodes ricinus* Tick-Borne Pathogens. Appl. Environ. Microbiol..

[94] Sekeyová Z., Fournier P.E., Řeháček J., Raoult D. (2000). Characterization of a New Spotted Fever Group *Rickettsia* Detected in *Ixodes ricinus* (Acari: Ixodidae) Collected in Slovakia. J. Med. Entomol..

[95] Christova I., van de Pol J., Yazar S., Velo E., Schouls L. (2003). Identification of *Borrelia burgdorferi* sensu lato, *Anaplasma* and *Ehrlichia* Species, and Spotted Fever Group Rickettsiae in Ticks from Southeastern Europe. Eur. J. Clin. Microbiol. Infect. Dis..

[96] Jado I., Oteo J.A., Aldámiz M., Gil H., Escudero R., Ibarra V., Portu J., Portillo A., Lezaun M.J., García-Amil C. (2007). *Rickettsia monacensis* and Human Disease, Spain. Emerg. Infect. Dis..

[97] Barradas P.F., Mesquita J.R., Mateus T.L., Ferreira P., Amorim I., Gärtner F., de Sousa R. (2021). Molecular Detection of *Rickettsia* spp. in Ticks and Fleas Collected from Rescued Hedgehogs (*Erinaceus europaeus*) in Portugal. Exp. Appl. Acarol..

[98] Barradas P.F., Mesquita J.R., Ferreira P., Amorim I., Gärtner F. (2020). Detection of Tick-Borne Pathogens in *Rhipicephalus sanguineus* sensu lato and Dogs from Different Districts of Portugal. Ticks Tick Borne Dis..

[99] Castillo-Contreras R., Magen L., Birtles R., Varela-Castro L., Hall J.L., Conejero C., Aguilar X.F., Colom-Cadena A., Lavín S., Mentaberre G. (2021). Ticks on Wild Boar in the Metropolitan Area of Barcelona (Spain) Are Infected with Spotted Fever Group Rickettsiae. Transbound. Emerg. Dis..

[100] Ortuño A., Sanfeliu I., Nogueras M.M., Pons I., López-Claessens S., Castellà J., Antón E., Segura F. (2018). Detection of *Rickettsia massiliae*/Bar29 and *Rickettsia conorii* in Red Foxes (*Vulpes vulpes*) and Their *Rhipicephalus sanguineus* Complex Ticks. Ticks Tick Borne Dis..

[101] Márquez F.J., Rodríguez-Liébana J.J., Soriguer R.C., Muniaín M.A., Bernabeu-Wittel M., Caruz A., Contreras-Chova F. (2008). Spotted Fever Group *Rickettsia* in Brown Dog Ticks *Rhipicephalus sanguineus* in Southwestern Spain. Parasitol. Res..

[102] Santos A.S., Santos-Silva M.M., Santos A.S., Santos-Silva M.M. (2018). *Ixodes ventalloi* Gil Collado, 1936: A Vector Role to Be Explored. Vectors Vector-Borne Zoonotic Dis..

[103] Raoult D., Fournier P.E., Abboud P., Caron F. (2002). First Documented Human *Rickettsia aeschlimannii* Infection. Emerg. Infect. Dis..

[104] Oteo J.A., Portillo A., Blanco J.R., Ibarra V., Pérez-Martínez L., Izco C., Pérez-Palacios A., Jiménez S. (2005). Low Risk of Developing Human *Rickettsia aeschlimannii* Infection in the North of Spain. Ann. N. Y. Acad. Sci..

[105] Portillo A., Santibáñez P., Santibáñez S., Pérez-Martínez L., Oteo J.A. (2008). Detection of *Rickettsia* spp. in *Haemaphysalis* Ticks Collected in La Rioja, Spain. Vector Borne Zoonotic Dis..

[106] Fernández-Soto P., Encinas-Grandes A., Pérez-Sánchez R. (2003). *Rickettsia aeschlimannii* in Spain: Molecular Evidence in *Hyalomma marginatum* and Five Other Tick Species That Feed on Humans. Emerg. Infect. Dis..

[107] Moerbeck L., Vizzoni V.F., Machado-Ferreira E., Cavalcante R.C., Oliveira S.V., Soares C.A.G., Amorim M., Gazêta G.S. (2016). *Rickettsia* (Rickettsiales: Rickettsiaceae) Vector Biodiversity in High Altitude Atlantic Forest Fragments Within a Semiarid Climate: A New Endemic Area of Spotted-Fever in Brazil. J. Med. Entomol..

[108] Fournier P.E., el Karkouri K., Leroy Q., Robert C., Giumelli B., Renesto P., Socolovschi C., Parola P., Audic S., Raoult D. (2009). Analysis of the *Rickettsia africae* Genome Reveals That Virulence Acquisition in *Rickettsia* Species May Be Explained by Genome Reduction. BMC Genom..

[109] Pérez-Pérez L., Portillo A., Allegue F., Zulaica A., Oteo J.A., Caeiro J.L., Fabeiro J.M. (2010). Dermacentor-Borne Necrosis Erythema and Lymphadenopathy (DEBONEL): A Case Associated with *Rickettsia rioja*. Acta Derm. Venereol..

[110] Gortázar C., Barroso-Arévalo S., Ferreras-Colino E., Isla J., de la Fuente G., Rivera B., Domínguez L., de la Fuente J., Sánchez-Vizcaíno J.M. (2021). Natural SARS-CoV-2 Infection in Kept Ferrets, Spain. Emerg. Infect. Dis..

